# The Economic Burden of Gastric Cancer: A Systematic Review

**DOI:** 10.1002/hsr2.72060

**Published:** 2026-04-14

**Authors:** Mohadeseh Ghanbari‐Jahromi, Najmeh Bordbar, Faride Sadat Jalali

**Affiliations:** ^1^ Research Center for Social Determinants of Health Jahrom University of Medical Sciences Jahrom Iran; ^2^ Health Human Resources Research Center, School of Health Management and Information Sciences Shiraz University of Medical Sciences Shiraz Iran

**Keywords:** cancer, economic burden, gastric, review, stomach

## Abstract

**Background and Aims:**

Gastric cancer is the fifth most common cancer, as well as the fourth leading cause of cancer‐related mortality. It significantly burdens patients, families, and the healthcare system. Considering this large economic impact, this review study aims to evaluate the total economic burden of gastric cancer.

**Methods:**

An extensive search was conducted in PubMed, Web of Science, ScienceDirect, Scopus, and ProQuest databases for all original research articles that evaluated/reported the economic burden of gastric cancer. No timing restrictions and studies up to December 2024 were included. The inclusion criteria were defined using the PICOTS model, and the quality of the selected studies was assessed using the Drummond checklist by two independent researchers. Data from each study were extracted using a standardized data extraction form.

**Results:**

The cost data originated from 17 studies of gastric cancer. Most of the studies were performed in Asia, and the societal perspective was the most common approach for cost measurement. Direct medical costs would represent the most significant component of the economic burden of gastric cancer, the authors found. Chemotherapy was the dominant cost factor among these. In addition, indirect costs included a significant share of non‐medical costs, such as travel, lodging, and home care. These included indirect productivity loss above due to absenteeism, premature mortality, and time costs.

**Conclusion:**

This review consolidates evidence that the economic burden of gastric cancer is multifaceted and substantial, driven predominantly by direct medical costs like chemotherapy but significantly amplified by indirect productivity losses. The variation in healthcare systems and stage at diagnosis are critical factors shaping this economic impact, underscoring the potential for early detection and tailored economic interventions to mitigate this burden.

**Abbreviations**
CPI: consumer price index
GC: gastric cancer
HICs: high‐income countries
*H. pylori*
: *Helicobacter pylori*
LMICs: low‐ and middle‐income countries
WHO: World Health Organization

## 1 Introduction

Gastric cancer (GC) ranks as the fifth most prevalent cancer globally and stands as the fourth leading cause of cancer‐related mortality [1]. GC develops when abnormal cells in the stomach begin to grow and divide uncontrolled [2]. According to the latest report by the World Health Organization (WHO), 970,000 new cases of GC were diagnosed in 2022, with 660,175 deaths reported that same year [3]. According to studies by the International Agency for Research on Cancer in 2022, it is predicted that the number of patients with GC will reach 1.67 million by the end of 2045 [4].

With increasing diagnostic and therapeutic technologies, lifestyle changes, and expectations from the healthcare system, healthcare spending and out‐of‐pocket expenses for patients' families are increasing [5]. Additionally, there is evidence that cancer patients and their families are under considerable financial burdens [6, 7]. Moreover, cancer, particularly GC, places a substantial financial burden on both households and the healthcare system. Beyond the direct impact of the disease, it also contributes to economic losses by reducing productivity and overall output. From a societal standpoint, these costs are considerable, accounting for 5%–10% of total healthcare expenditures in many developed nations [8].

It is regarded as a severe disease in terms of management and treatment, with associated costs being nearly 10 times higher than those of non‐cancerous conditions, particularly as the disease progresses [9]. Research conducted in 2016 estimated the total cost and economic burden of GC in Kohgilooyeh and Boyer‐Ahmad province, Iran, at $436,237, of which the majority was direct medical costs (59%) [9]. According to a study by Jalilian et al. in Iran (2019), the average direct medical cost was estimated to be $3288.02, and the average direct non‐medical cost was $377.54 for GC. The average total cost was also $4170.97, with 35.5% of the cost being borne by the patient [7]. Zhang et al. showed that the average total direct cost per patient was $9899 (medical cost 91.2%, non‐medical cost 8.8%), and the out‐of‐pocket cost of a patient with newly diagnosed GC was $5368 per year [6]. Solanki et al. found that the average cost of care per inpatient for GC increased from $21710 in 2001 to $24706 in 2011 [10]. Another study in China showed that the annual cost of illness for patients with gastric and esophageal cancer was $10,449 in urban areas and $2927 in rural areas, respectively [11].

Timely estimation of the costs of cancer care helps assess the population's overall health in the current healthcare system. This assessment is crucial for national cancer programs and policies, as it supports the development of effective strategies to reduce the financial burden on families and society [6]. Therefore, this study systematically compares the costs of GC in low‐ and middle‐income countries (LMICs) and high‐income countries (HICs) based on recent research on the cost of this disease, which has not been covered in previous reviews. This study examines the global and regional economic impact of GC. It comprehensively analyzes the social, healthcare, and individual perspectives, disaggregating this disease's direct and indirect costs. The findings of this pioneering review can serve as a valuable resource for cost‐effectiveness analyses of GC‐related diseases and for guiding the development and implementation of clinical guidelines.

## 2 Methods

This study conducted a systematic review in accordance with the Preferred Reporting Items for Systematic Reviews and Meta‐Analyses (PRISMA) guideline. The review utilized articles related to the economic burden of GC based on the PICOTS model to establish inclusion criteria, a standard framework recommended for structuring systematic review questions [12, 13]. The study population comprised patients diagnosed with GC, while interventions encompassed various clinical treatments. No specific limitations were placed on comparators. The outcomes assessed included both direct and indirect costs associated with GC. The review considered studies published up to December 2024.

The development of the search strategy, including the identification of clinical and economic terms, was conducted in collaboration with a medical librarian to ensure its robustness and comprehensiveness across all selected databases.

The study design focused on cost‐benefit analyses that gathered cost‐related data from GC patients. A societal perspective was adopted for cost estimation, as it provides a comprehensive evaluation by incorporating all direct (medical and non‐medical) and indirect costs. This approach enables a thorough assessment of the financial impact of GC, making it the preferred perspective over others. Unlike narrower approaches, the societal perspective examines not only the economic effects on the patient or healthcare provider but also the broader implications for families and the healthcare system as a whole [14]. Relevant studies were identified and retrieved using a structured search strategy (Table 1).

**Table 1:** Search strategy for the economic burden of gastric cancer literature review.

Database	Search string	*N*
Scopus	(TITLE (neoplasm OR cancer) AND TITLE (stomach OR gastric) AND TITLE (cost OR expenditure OR “cost of illness” OR “cost analysis” OR economics OR “burden of illness” OR “economic burden” OR “illness burden” OR “direct cost” OR “indirect cost” OR “financial burden” OR expense)) AND (LIMIT‐TO (LANGUAGE, “English”))	156
PubMed	Search: ((neoplasm[Title] OR cancer[Title]) AND (Stomach[Title] OR Gastric[Title])) AND (cost[Title] OR expenditure[Title] OR “cost of illness”[Title] OR “cost analysis”[Title] OR economics[Title] OR “burden of illness”[Title] OR “economic burden”[Title] OR “illness burden”[Title] OR “direct cost”[Title]c OR “indirect cost”[Title] OR “financial burden”[Title] OR expense[Title]) Filters: English	122
WOS	neoplasm OR cancer (Title) and Stomach OR Gastric (Title) and cost OR expenditure OR “cost of illness” OR “cost analysis” OR economics OR “burden of illness” OR “economic burden” OR “illness burden” OR “direct cost” OR “indirect cost” OR “financial burden” OR expense (Title) and English (Languages)	231
ProQuest	title(neoplasm OR cancer) AND title (Stomach OR Gastric) AND title(cost OR expenditure OR “cost of illness” OR “cost analysis” OR economics OR “burden of illness” OR “economic burden” OR “illness burden” OR “direct cost” OR “indirect cost” OR “financial burden” OR expense) Limits applied	49
Science Direct	title(neoplasm OR cancer) AND title (Stomach OR Gastric) AND title(cost OR expenditure OR “cost of illness” OR “cost analysis” OR economics OR “burden of illness” OR “economic burden” OR “illness burden” OR “direct cost” OR “indirect cost” OR “financial burden” OR expense)	62
Total		620

**Figure 1:**
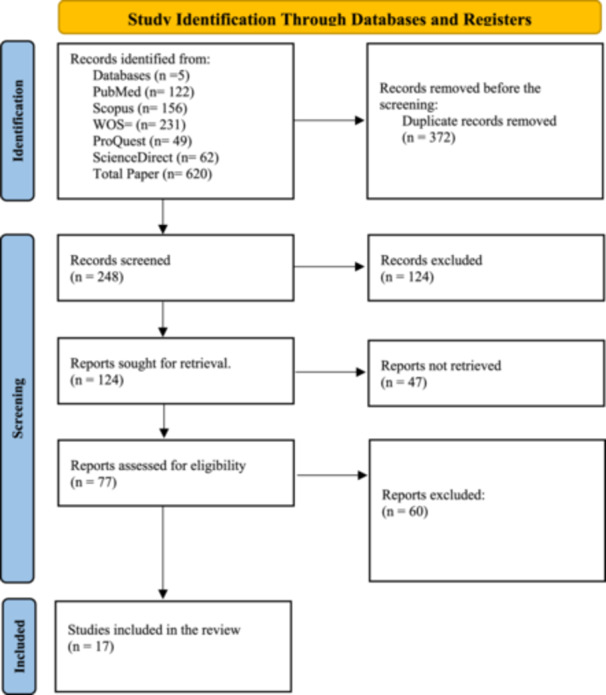
PRISMA flow diagram illustrating the systematic review process.

The selection of relevant studies was carried out in multiple stages. Initially, 620 articles were identified across all databases. Reference management and duplicate removal were performed using EndNote 20. After eliminating duplicate entries, 248 articles remained for further review. The subsequent screening of titles and abstracts was conducted independently by two researchers using the web‐based platform Rayyan (Qatar Computing Research Institute). After assessing titles and abstracts, 171 articles were excluded, leaving 77 for full‐text evaluation. Drummond's checklist was utilized to assess the quality of the studies, with modifications made to exclude sections unsuitable for costing studies. The results of the quality assessment are presented in Table S1. Ultimately, the research team selected 17 articles for inclusion (Figure 1).

Two researchers conducted the selection process independently, with a third researcher resolving any discrepancies through consensus. In the final stage, a standardized data extraction form was used to collect relevant information from each study. The form was designed in Microsoft Word (version 2021), and all extracted data were compiled and managed in Microsoft Excel 2021 to ensure accuracy and facilitate analysis. Extracted data included publication year, research location, sample size, study perspective, costing methodology, types of costs assessed, and average direct medical, direct non‐medical, and indirect costs. Since the studies were conducted in various countries and across different years, cost figures were adjusted for comparability using the Consumer Price Index (CPI) data obtained from the World Bank [15].

## 3 Results

After evaluating the articles using the PICOTS model, data from 17 studies on the costs of GC patients were extracted. The findings of this review are summarized in Tables 2 and 3.

**Table 2:** Studies on the economic burden of gastric cancer.

N	Author	Year	Place	Participants	Study perspective	Approach	Measured cost type
1	Abraham [16]	2020	US	392 patients with advanced metastatic gastric cancer	Societal perspective	Bottom‐up	Direct costs
2	Castro [17]	2017	US	524 patients with gastric adenocarcinoma	Patient perspective	Bottom‐up	Direct costs and direct non‐medical costs
3	Chen [18]	2017	China	176 advanced gastric cancer	Chinese healthcare payer	Bottom‐up	Direct medical costs, direct non‐medical costs, and indirect costs
4	Eghdami [9]	2019	Iran	110 gastric cancer patients	Patients' perspective	Bottom‐up	Direct medical costs, direct non‐medical costs, and indirect costs
5	Gourzoulidis [19]	2021	Greece	—	Public payer perspective	Top‐down	Total cost
6	Haga [20]	2013	Japan	127,690 stomach cancer	—	Bottom‐up	Direct costs and indirect costs
7	He [21]	2013	China	188 gastric cancer (stage 2 and 3)	Societal perspective	Bottom‐up	Direct medical costs, direct non‐medical costs
8	Hess [22]	2016	US	5257 patients with gastric cancer	Third‐party payer perspective and patient perspective	Bottom‐up	Direct medical costs
9	Hong [23]	2017	Taiwan	82 advanced gastric cancer	Societal perspective	Bottom‐up	Direct medical costs, direct non‐medical costs, and indirect costs
10	Izadi [24]	2016	Iran	160 patients with stomach cancer	Third payer's patient perspective	Bottom‐up	Direct medical costs
11	Jalilian [7]	2019	Iran	102 with gastric cancer at the period of the first 6 months after diagnosis	Perspective of society	Bottom‐up	Direct medical costs, Direct non‐medical costs, and Indirect costs
12	Karve [25]	2015	US	2583 elderly patients diagnosed with advanced stage gastric cancer	Third payer's perspective	Bottom‐up	Direct medical costs
13	Zhoua [26]	2017	Hong Kong	58 advanced gastric cancer	Healthcare societal perspectives.	Bottom‐up	Direct medical costs and indirect costs
14	Mohammadpour [27]	2020	Iran	449 patients with gastric cancer	—	Bottom‐up	Direct medical costs
15	Quintana [28]	2017	Mexico	180 advanced and metastatic gastric cancer	Payer perspective	Bottom‐up	Direct medical costs
16	Saito [29]	2017	Japan	Patients with advanced gastric cancer	Healthcare payer's perspective	Bottom‐up	Direct medical costs
17	Bilici [30]	2017	Turkey	8865	Healthcare payer perspective	Bottom‐up	Direct medical costs, direct non‐medical costs, and indirect costs

**Table 3:** Average direct and indirect costs of the economic burden of gastric cancer (2023 USD).

N	First author	Direct medical costs			Direct non‐medical costs			Indirect costs			Total cost	
		Per patient (Mean)	Total	Constituents	Per patient (Mean)	Total	Most constituents	Per patient (Mean)	Total	Most constituents	Per Patient (Mean)	Total
1	Abraham [16]	617.42	242,030.54	Inpatient admissions (32.2%), emergency room visits (1.6%), Outpatient services (66.2%)^a^, physician office visits, Physician other services, procedures, imaging, tests—lab, tests —other b, durable medical equipment, medication and related services, PT/OT/speech, pharmacy prescriptions	—	—	—	—	—	—	—	—
2	Castro [17]	4536.47	2,377,124.85	Hospital admission (51.8%)^a^, chemotherapy (29.2%), radiotherapy (9.3%), surgery (9.7%)^b^	—	—	—	—	—	—	—	—
3	Chen [18]	257.95	45,400.78	Medicine (89.0%), outpatient services (1.4%), tests (6.6%)	552.41	97,225.04	Travel (1.2%)	855.16	150,508.17	Absenteeism (1.9%)	—	—
4	Eghdami [9]	16,281.85	1,790,768.40	Physicians and oncologists' visits (8.57%), emergency department visits (2.53%), chemotherapy (14.14%)^a^, surgical services (9.50%), laboratory tests (11.45%), ultrasonography (1.78%), MRI (1.01%), CT scan (1.18%), radiology (1.37%), radiotherapy (2.09%), endoscopy (3.26%), colonoscopy (1.09%), transfer by ambulance (0.07%)^b^, patient hospitalization (6.64%), medications (35.32%)	3616.65	397,907.73	Transportation (30.93%)^a^, accommodation (21.94%), meals for the patient and relatives (15.99%), patient consumables (14.20%)^b^, accommodation for people who visit the patient at his/her home (16.95%)	7579.72	833,769.50	Patients' absence from work and daily activities caused by illness (43.74%)^a^, absence of patients' families from work and daily activities caused by patient care (56.26%)^b^	27,478.23	3,022,445.63
5	Gourzoulidis [19]	—	—	—	—	—	—	—	—	—	—	5091.83
6	Haga [20]	15,195.37	1,940,296.6 (22.77%)	—	—	—	—	51,533.68	6,580,335,806.06	Mortality (72.38%)^a^, Morbidity (4.85%)^b^	—	—
7	He [21]	18,760.97	3,533,226.51	Chemotherapy (86.4%)^a^, drugs, hospital bed cost (0.9%)^b^, tests (9.8%), venous access (2.9%)	70.76	13,288.97	Travel (43.2%)	91.51	17,422.56	Time (56.8٪)	—	—
8	Hess [22]	313.72	681,434.27	—	—	—	—	—	—	—	—	—
9	Hong [23]	31,430.40	2,577,293.07	Inpatient costs (28.64%), outpatient costs (11.80%)^b^, Chemotherapy (59.56%)^a^	5552.14	455,275.30	Caregiver costs (84.96%)^a^ Transportation (15.04%)^b^	180,451.02	14,796,983.4	Mortality (95.6%)^a^, morbidity (4.4%)^b^	—	—
10	Izadi [24]	19,283.19	3,085,311.02	Medications cost (56.3%)^a^, chemotherapy cost (9.2%), surgery cost (16.7%), radiotherapy cost (6.9%), tests/radiology cost (5.9%), visit cost (5.0%)^b^	—	—	—	—	—	—	—	—
11	Jalilian [7]	21,093.41	2,151,527.33	Physician visits (1.19%), emergency visits (0.34%), diagnostic services (3.45%), medicine (4.53%), surgery (2.65%), chemotherapy (5.15%)^a^, complementary treatments (0.61%), medical equipment and supplies (0.26%)^b^	2422.01	247,044.61	Travel cost	3242.32	330,716.79	Lost productivity	26,757.73	2,729,295.15
12	Karve [25]	173,847	242,485,158.75	Radiation therapy (16.1%), biologic therapy (1.1%)^b^, chemotherapy, overall (37.3%)^a^, chemotherapy, drugs (29.0%), chemotherapy, administration (8.3%)	—	—	—	—	—	—	—	—
13	Zhoua [26]	4060.27	235,495.45	Medication (27.6%)^a^, day ward/inpatient (7.3%) visits^b^, laboratory tests (13.9%), x‐rays and scans (9.3%), outpatient visits (13.4%)	76.79	4454.21	Travel (8.8%)	160.50	9309.38	Time lost (12%)	4297.56	249,258.38
14	Mohammadpour [27]	10,451.62	4,692,777.43	Endoscopy (4.87%), biopsy (0.10%) b, surgery (11.27%), gerafy (4.31%), scan (1.28%), radiotherapy (3.75%), laboratory (18.05%), chemotherapy (2.12%), hospitality (54.22%)^a^	—	—	—	—	—	—	—	—
15	Quintana [28]	22,842.70	4,111,685.79	Drug acquisition (14.1%), administration (47.6%)^a^, adverse events (0.4%) b, radiotherapy (0.7%), hospitalization (inpatient) (15.0%), outpatient visits (4.1%), supportive care (16.3%)	—	—	—	—	—	—	—	—
16	Saito [29]	—	—	—	—	—	—	—	—	—	—	52,994.84
17	Bilici [30]	61,561.17	545,739,812.84	Outpatient cost (3.3%), screening and laboratory costs (6%), hospitalization and medical interventions (14.2%), medication and application costs (36.8%), side effects and complication cost (39.6%)	15,353.68	136,110,402.25	—	753,475.67	6,679,561,836.29	—	—	—

^a^ Most.

^b^ Least.

Table 2 presents the studies' characteristics, including the research year and location, sample size, costing methodologies, and the types of costs assessed. As shown in Table 2, the majority of studies were conducted in Asia (64.71%), followed by the United States (29.41%), with one study from Europe (5.88%). The largest sample size included 127,690 participants. The societal perspective was the most commonly used approach for cost measurement. The predominant costing method employed in most studies was the population‐based approach (both prevalence‐based and bottom‐up). Most studies focused on direct costs, and nearly half included indirect costs. As outlined in Table 3, four studies (23.53%) identified direct medical costs as the primary contributor to the economic burden of GC. In comparison, three studies (17.64%) highlighted indirect costs as the main factor driving the financial burden.

Among the 15 studies reporting direct medical costs, six (40%) identified chemotherapy costs as the primary component of these types of costs. Of the seven studies that reported direct non‐medical costs, five (71.4%) highlighted travel and accommodation costs, while two (28.5%) focused on home care costs as the most significant contributors. Additionally, eight studies examined the components of indirect costs. Among them, two studies (25%) cited absenteeism and premature death costs, and one study (12.5%) identified the cost of reduced productivity and time as the main drivers of indirect costs.

Table 3 indicates that the highest and lowest mean direct medical costs were observed in the United States ($173,847) and China ($25,795), respectively. Regarding direct non‐medical costs, the highest mean was reported in Turkey ($15,353.68) and the lowest in China ($70.76). For indirect costs, Turkey reported the highest average cost ($753,475.67), while China recorded the lowest ($91.50).

A detailed breakdown of cost components, expressed as a percentage of the total cost category, is provided for each study in Table 3. For instance, in Hong et al., chemotherapy constituted the largest share of direct medical costs (59.56%), followed by inpatient costs (28.64%). In Jalilian et al. study, physician visits, emergency visits, and diagnostic services accounted for 1.19%, 0.34%, and 3.45% of direct medical costs, respectively. The analysis reveals considerable variation in the primary cost drivers across different healthcare systems and study perspectives. Chemotherapy was a major cost component in several studies, while hospitalization and medication costs dominated in others. Among indirect costs, mortality costs associated with productivity loss were the most significant factor in studies that reported them.

## 4 Discussion

GC is among the most prevalent cancers globally and is ranked as the fifth leading cause of cancer‐related deaths worldwide in 2022 [31, 32, 33]. *Helicobacter pylori* (*H. pylori*) infection is identified as the most important biological risk factor for GC, accounting for 78% of all cases [34]. The cost of treatment is very high and causes tremendous strain on limited healthcare resources [35]. Insights that can improve the functioning of health organizations at all levels can be gained by assessing the costs of illness [27]. This study aimed to provide an overview of the economic burden of GC through a systematic review.

The costs incurred by the system were categorized into two primary groups: direct and indirect costs. Direct costs were further broken down into medical and non‐medical costs. Medical costs refer to the financial outlays related to healthcare services, such as diagnosis, treatment, and rehabilitation [9, 36, 37]. In the studies examined in this research, direct medical costs were identified as the primary contributor to the economic burden faced by GC patients [7, 9, 18, 21], and chemotherapy costs were the highest component of direct medical costs [7, 16, 22, 23, 25, 38]. After that, hospitalization costs [17, 26, 27] and medication costs [5, 9] were the highest. The elevated expenses associated with Chemotherapy for GC patients may be attributed to the high prices of these medications. Karve et al., in their study in the USA, found that expenses associated with Chemotherapy, including both medication and administration costs, represented 68% of the overall treatment costs related to GC [25]. In the study conducted by Castro et al. in Panama, the most significant expenses were linked to hospital admissions, while chemotherapy represented the second‐largest cost [17]. In the study conducted by Izadi et al. in Iran, it was observed that the most significant proportion of costs was associated with surgical procedures in Stage I.

In contrast, medication costs were predominant in Stages II, III, and IV. Furthermore, the costs for medication and Chemotherapy escalated from Stage I to Stage IV, whereas the expenses related to surgery, radiation therapy, diagnostic tests, and consultations diminished [5]. Therefore, it seems that the stage of GC is influential in estimating the costs and types of fees imposed on the health system [6].

The results of this study revealed that the primary factors contributing to direct non‐medical costs include costs related to patients' travel, transportation, and accommodation [7, 9, 18, 21, 26], caregiver costs [23], and lodging for individuals visiting the patient at their home [9]. The average non‐medical costs incurred by non‐native patients exceeded those of native patients. The absence of relevant medical facilities in the cities where individuals reside, coupled with insufficient public transportation for patients needing to travel to the capital for essential services and medical treatments, contributes to elevated transportation expenses [7, 9, 39]. The study by Jalilian et al. in Iran found that the overall mean cost associated with GC was more significant for patients who delayed their treatment due to the considerable distance from the treatment center and financial constraints, as opposed to those who did not experience such delays [7].

Also, in this study, the indirect cost was related to the absenteeism of patients and their families from work and daily activities [9, 18], mortality, and premature death [23, 40], the cost of reduced productivity and loss of time for patients and caregivers [7, 26]. The likelihood of developing stomach cancer escalates with advancing age, with over 80% of cases diagnosed in individuals aged between 60 and 80 years. Consequently, members of this age group must be accompanied by at least one family member when visiting medical facilities for necessary services and treatment. This requirement has resulted in extended absences from work and daily activities for patients' families, leading to significant financial implications [1, 9]. The time lost by patients and their families due to the Absence of a regular work schedule stemming from illness and treatment, which includes the time spent traveling to and from medical care, waiting for appointments, and similar activities, is regarded as an indirect economic consequence. Consequently, the loss of time is a significant aspect of the overall burden of illness experienced by both patients and their caregivers [6], and in the study conducted by Hong et al. in Taiwan, mortality costs, accounting for 77.3%, emerged as the predominant factor contributing to the overall burden. This was followed by direct medical expenses, representing 16.3% [23].

The study found that the highest average direct medical costs were reported in the United States, direct non‐medical and indirect costs in Turkey, and the lowest average costs were reported in China. GC is characterized by poor survival rates and is linked to the absence of established treatment protocols, especially after the first‐line therapy [22]. Variations in average costs among studies conducted in various countries may be attributed to differing definitions of care types, as each report encompassed a diverse array of services in their cost assessments [17]. In addition, age, gender, length of stay [7, 27, 40], public or private hospital, education, employment status, stage of diseases, type of carcinoma, type of insurance scheme [5, 6, 7], delays in treatment attributed to the considerable distance from the treatment center, delays due to financial and economic obstacles [7], providing systems, and medical technology could affect the cost of illness in different health systems [40].

A recently published systematic review by Sharma et al. also examined the economic impact of gastric cancer. Their review identified 19 economic evaluations, of which four were cost‐of‐illness studies. While their work focused specifically on the cost‐effectiveness of second‐line or later treatments for advanced disease, our study provides a comprehensive analysis of the cost‐of‐illness across all disease stages. Both reviews confirm the substantial economic burden of gastric cancer. However, our findings, which are based on a larger number of cost‐of‐illness studies (*n* = 17), offer a more detailed breakdown of cost components and their primary drivers, such as chemotherapy within direct medical costs. The variation in cost structures observed in our review aligns with the methodological heterogeneity reported by Sharma et al. across different healthcare systems [41].

A comparative perspective with other major cancer types further illuminates the distinctive economic and clinical challenges posed by gastric cancer. An analysis suggests that while the absolute costs of GC may be lower than those of malignancies characterized by prolonged targeted therapies, such as breast or colorectal cancer [42, 43, 44], its economic impact is particularly severe when considered in light of its typically late‐stage diagnosis and poor prognosis. The significant variation in cost structures across different countries is less a reflection of clinical practice and more a direct consequence of fundamental differences in healthcare financing and delivery systems [45, 46, 47]. In insurance‐based models, for instance, high pharmaceutical costs are a primary driver of expenditures [48, 49], whereas in tax‐funded systems, inpatient care and indirect productivity losses constitute a larger proportion of the total burden [50]. These system‐level determinants are linked to disparities in patient access to care and survival rates. Consequently, managing the economic burden of GC necessitates policy interventions that are specifically tailored to address the unique inefficiencies and barriers within a given health system, moving beyond a one‐size‐fits‐all approach.

### 4.1 Strengths and Limitations

This study stands out for its comprehensive analysis of the economic burden of GC, consolidating data from 17 studies worldwide and adopting a societal perspective to capture both direct and indirect costs. Its global relevance, methodological rigor, and actionable insights for policymakers make it a valuable contribution to the literature, particularly as the first systematic review to address this topic comprehensively. However, limitations include the geographic concentration of studies in Asia, variability in cost measurement methodologies, and the time lag in data collection. Additionally, the protocol for this systematic review was not prospectively registered, which may introduce potential hindsight bias in the study selection process. Furthermore, the review does not address intangible costs like emotional distress, lacks outcome‐based cost‐effectiveness analysis, and excludes non‐English studies, which may limit the generalizability of its findings.

## 5 Conclusion

The results showed that GC causes high direct and indirect costs for patients. Also, these costs impose a significant economic burden on families and the health system. Considering the mentioned points, patients and the healthcare system face significant financial challenges related to this disease. Consequently, policymakers and decision‐makers must investigate the economic ramifications of GC, one of the most prevalent cancer types. Such an investigation yields important insights informing resource distribution and cost management initiatives. Implementing early detection and effective treatment protocols based on evidence‐based guidelines, conducting laboratory tests for *Helicobacter pylori* (the primary cause of GC) in all patients with gastrointestinal conditions, and offering educational programs on GC prevention from a young age can alleviate the economic impact of GC.

## Author Contributions

F.S.J., N.B., and M.G.H‐J did the search, screening, and data extraction, raised the research idea, and supervised all phases of the research. All authors have equal contributions in drafting and reviewing the manuscript. All authors have read and approved the final manuscript.

## Funding

The authors have nothing to report.

## Ethics Statement

This is not applicable as the study is based on extracting data from published articles.

## Conflicts of Interest

The authors declare no conflicts of interest.

## Transparency Statement

The corresponding author, Faride Sadat Jalali, affirms that this manuscript is an honest, accurate, and transparent account of the study being reported; that no important aspects of the study have been omitted; and that any discrepancies from the study as planned (and, if relevant, registered) have been explained.

## Supporting information

Supporting File

## Data Availability

The study is based on extracting data from published articles; all data are included in the report.
