# Supplementary material for: The Economic Burden of Gastric Cancer: A Systematic Review
Source: Health Sci Rep. 2026 Apr 14;9(4):e72060. doi: 10.1002/hsr2.72060 (PMC13077669; doi:10.1002/hsr2.72060)
Supplement: Supplementary file 1 — Supporting File [file HSR2-9-e72060-s001.docx]

**Table S1: Quality assessment of included economic evaluations using the Drummond 10-item checklist**

| **Article (Author/Year)** | **Research question well defined?** | **Comprehensive description of alternatives?** | **Effectiveness of program established?** | **Important & relevant costs & consequences for each alternative identified?** | **Costs & consequences measured accurately & appropriately?** | **Costs & consequences valued credibly?** | **Costs & consequences adjusted for differential timing?** | **Incremental analysis of costs & consequences performed?** | **Allowance made for uncertainty in estimates?** | **Presentation & discussion of study results include all issues of concern to users?** | **Quality Category** |
| --- | --- | --- | --- | --- | --- | --- | --- | --- | --- | --- | --- |
| Abraham, 2020 | Yes | No | No | Yes | Yes | Yes | No | No | Yes | Yes | Good |
| Castro, 2017 | Yes | Yes | Yes | Yes | Yes | Yes | No | Yes | Yes | Yes | Good |
| Chen, 2017 | Yes | Yes | Yes | Yes | Yes | Yes | Yes | Yes | Yes | Yes | Good |
| Eghdami 2019 | Yes | Yes | Yes | Yes | Yes | Yes | No | No | Yes | Yes | Good |
| Gourzoulidis 2021 | Yes | Yes | Yes | Yes | Yes | Yes | No | No | Yes | Yes | Good |
| Haga 2013 | Yes | Yes | Yes | Yes | Yes | Yes | No | No | Yes | Yes | Good |
| He 2013 | Yes | Yes | Yes | Yes | Yes | Yes | Yes | No | Yes | Yes | Good |
| Hess 2016 | Yes | Yes | Yes | Yes | No | Yes | Yes | No | Yes | Yes | Good |
| Hong 2017 | Yes | Yes | Yes | Yes | Yes | Yes | No | No | Yes | Yes | Good |
| Izadi 2016 | Yes | Yes | Yes | Yes | Yes | Yes | Yes | Yes | Yes | Yes | Good |
| Jalilian 2019 | Yes | No | Yes | Yes | Yes | Yes | No | No | Yes | Yes | Good |
| Karve 2015 | Yes | No | Yes | Yes | Yes | Yes | Yes | No | Yes | Yes | Good |
| Zhou 2017 | Yes | Yes | Yes | Yes | Yes | Yes | No | Yes | Yes | Yes | Good |
| Mohammadpour 2020 | Yes | No | Yes | Yes | No | Yes | No | Yes | Yes | Yes | Good |
| Saito 2017 | Yes | Yes | Yes | Yes | Yes | Yes | No | No | Yes | Yes | Good |
| [Bilici](https://www.valueinhealthjournal.com/article/S1098-3015(17)30513-2/fulltext) 2017 | Yes | Yes | Yes | Yes | Yes | Yes | No | No | Yes | Yes | Good |
